# Genome-wide identification of *YABBY* gene family and its expression pattern analysis in *Astragalus mongholicus*

**DOI:** 10.1080/15592324.2024.2355740

**Published:** 2024-05-22

**Authors:** Jiamei Wang, Zhen Wang, Panpan Wang, Jianhao Wu, Lingyang Kong, Lengleng Ma, Shan Jiang, Weichao Ren, Weili Liu, Yanli Guo, Wei Ma, Xiubo Liu

**Affiliations:** a Equipment Department, Heilongjiang University of Chinese Medicine, Harbin, China; b Pharmacy of College, Heilongjiang University of Chinese Medicine, Harbin, China; c College of Jiamusi, Heilongjiang University of Chinese Medicine, Jiamusi, China

**Keywords:** *Astragalus mongholicus*, *YABBY* gene family, phylogenetic, expression pattern, bioinformatics

## Abstract

During plant growth and development, the *YABBY* gene plays a crucial role in the morphological structure, hormone signaling, stress resistance, crop breeding, and agricultural production of plant lateral organs, leaves, flowers, and fruits. *Astragalus mongholicus* is a perennial herbaceous plant in the legume family, widely used worldwide due to its high medicinal and edible value. However, there have been no reports of the *YABBY* gene family in *A. mongholicus*. This study used bioinformatics methods, combined with databases and analysis websites, to systematically analyze the *AmYABBY* gene family in the entire genome of *A. mongholicus* and verified its expression patterns in different tissues of *A. mongholicus* through transcriptome data and qRT-PCR experiments. A total of seven *AmYABBY* genes were identified, which can be divided into five subfamilies and distributed on three chromosomes. Two pairs of *AmYABBY* genes may be involved in fragment duplication on three chromosomes. All AmYABBY proteins have a zinc finger YABBY domain, and members of the same group have similar motif composition and intron – exon structure. In the promoter region of the genes, light-responsive and MeJa-response *cis*-elements are dominant. *AmYABBY* is highly expressed in stems and leaves, especially *AmYABBY1*, *AmYABBY2*, and *AmYABBY3*, which play important roles in the growth and development of stems and leaves. The *AmYABBY* gene family regulates the growth and development of *A. mongholicus*. In summary, this study provides a theoretical basis for in-depth research on the function of the *AmYABBY* gene and new insights into the molecular response mechanism of the growth and development of the traditional Chinese medicine *A. mongholicus*.

## Introduction

*Astragalus memeranaceus* (Fisch.) Bge. Var. *mongholicus* (Bge.) Hsiao is a perennial herbaceous plant of the family Leguminosae and is widely used worldwide due to its very high medicinal and food value.^1^ *A. mongholicus* is the basal plant of the Chinese medicine astragalus (ASTRAGALI RADIX), and its dried root is the medicinal part, which is used in more than 200 herbal formulas, with a history of application of more than 2,000 years.^2^ *A. mongholicus* is a tonic herb that is used to improve vital energy, strengthen the spleen, tranquilize the heart, moisten the intestines and act as a laxative, and astringe the yin to stop sweating.^3^ The chemical composition of the *A. mongholicus* complex mainly includes saponins, flavonoids, and polysaccharides. It is also rich in amino acids and trace elements.^4,5^ Pharmacological activities can improve the body’s immunity and number of scavenging free radicals and promote anti-inflammatory, anti-tumor, anti-diabetic, antioxidant, and other effects.^6,7^ The growth of *A. mongholicus* is a delicate process,^8^ and the study of the molecular mechanism of its growth and development is of great significance in guiding production in the face of an increasingly severe natural environment.

The *YABBY* gene family belongs to the zinc finger protein superfamily and is a plant-specific transcription factor.^9^ All YABBY members share two highly conserved structural domains characterized by an N-terminal zinc finger structural domain (C2-C2) and a C-terminal YABBY structural domain (helix – loop – helix).^10^ There are five subfamilies of *YABBY* genes, namely, CRC (CRABS CLAW), FIL (FILAMENTOUS FLOWER)/YAB3, INO (INNER NO OUTER), YAB2, and YAB5.^11^ *YABBY* genes play a crucial role in morphogenesis, including lateral organs, leaf, flower, and fruit development.^12^ The *YABBY* gene family is widely involved in plant life processes. Studies have shown that tomato *SlCRCa* is highly expressed in petals and stamens and is sensitive to gibberellin (GA) treatment. Overexpression of *SlCRCa* made tomato petals, stamens and fruits smaller, while knock-out *SlCRCa* showed the opposite phenotype.^13^ *INO* in *Arabidopsis thaliana* protects reproductive development by reducing iron load in developing seeds by inhibiting *NRAMP1* expression.^14^ Overexpression of the BcYAB3 gene in Chinese cabbage in *A. thaliana* can lead to downward curling of leaves, delayed bolting and flowering.^15^ It has been shown that FIL genes are associated with floral organ formation and leaf development,^16^ YAB2, YAB3 and YAB5 are specifically expressed in plant trophic tissues.^17,18^ In *A.thaliana*, the CRC gene, a member of the *YABBY* gene family, is involved in the development of nectaries and carpels, and the INO gene promotes the development of the external bead cover.^19^ Overexpression of the soybean *GmFILa* transcription factor in *A. thaliana* leads to changes in the dorsal and ventral polarity of the epidermal leaf tissues of the transgenic plants, and the apical meristem development is inhibited.^20^ Currently, reports on *YABBY* genes have been carried out in various plants; however, whether *YABBY* genes are involved in the growth and development of *A. mongholicus* remains largely unknown. Currently, a high-quality genome of *A. mongholicus* has been released,^21^ therefore, it is of great significance to mine the potential function of the *AmYABBY* gene in the growth and development of *A. mongolianus* based on whole-genome data and transcriptome data.

In this study, we performed genome-wide identification and characterization of the *A. mongholicus YABBY* gene family members of *A. mongholicus*. We also comprehensively analyzed the physicochemical properties, collinearity, phylogenetic evolution, gene structure, and *cis*-regulatory elements of the *AmYABBY* genes. In addition, the expression patterns of *AmYABBY* genes during the growth of *A. mongholicus* were comprehensively analyzed by transcriptome data combined with RT-qPCR experiments, which provided valuable information for screening candidate *YABBY* genes involved in the regulation of the growth and development of *A. mongholicus*.

## Materials and methods

### Plant material

The plant material, *A. mongholicus* seeds, was identified by the Wei Ma Research Institute of Heilongjiang University of Chinese Medicine. Soil-less cultivation was carried out in the light incubator of the Medicinal Molecular Laboratory of Heilongjiang University of Chinese Medicine. The nutrient solution used was an improved Hoagland nutrient solution (Coolaber Technology Co., Ltd., Beijing, China), with a light intensity of 2000–2500 Lux, a temperature of 25°C, and a light cycle of 16 hours (light)/8 hours (dark). When the seedlings of *A. mongholicus* were 40 days old, healthy plants with similar growth conditions were selected, and roots, stems, and leaves of the same plant were sampled. After collection, they were frozen with liquid nitrogen and stored in a −80°C ultra-low temperature refrigerator for further analysis.

### Data sources

The whole-genome sequence and annotation files of *A. mongholicus* were downloaded from the Glo-bal Pharmacopoeia Genome Database (GPGD)^22^ website (http://www.gpgenome.com/species/109). *A. thaliana* (GCA_000005425. 2), Malus domestica (GCF_002114115.1), *Codonopsis lanceolata* (GCA_013146195.2), and *O.sativa* (GCA_001433935.1) genome files and annotation files were downloaded from the NCBI database (https://www.ncbi.nlm.nih.gov/).

### *Identification of the* AmYABBY *gene family*

In order to identify all members of the *YABBY* gene family in the genome of *A. mongholicus*, all protein sequences in the genome of A. mongholicus were extracted using TBtools software^23^ and submitted to the Plant Transcription Factor Database (http://planttfdb.gao-lab.org/index.php) to obtain the *YABBY* gene candidate sequences, and the structural domains of the candidate sequences were confirmed using the CD-search (https://www.ncbi.nlm.nih.gov/cdd/) online tool. After removing redundant and structurally domain incomplete sequences, the final *AmYABBY* gene was obtained. Physicochemical properties of the AmYABBY protein, including molecular mass, isoelectric point, and hydrophobicity, were analyzed using the online analysis software ExPASy ProtParam (http://web.expasy.org/ProtParam). On another online platform (http://www.csbio.sjtu.edu.cn/bioinf/euk-multi-2/), *AmYABBY* protein subcellular localization results were used to obtain the prediction.

### Secondary and tertiary structure prediction of gene-encoded proteins

We used TBtools software to extract and obtain protein sequences of *A. mongholicus YABBY* gene family members and used the SOPMA (https://npsa-prabi.ibcp.fr/cgi-bin/npsa_automat.pl.page=npsa_sopma.html) online website to predict the *A. mongholicus* YABBY protein secondary structure. The tertiary structure of the *A. mongholicus* YABBY protein (https://swissmodel.expasy.org/) was constructed using the SWISS-MODEL online site.

### Chromosome location and evolution analysis

The chromosome location information of the *A. mongholicus YABBY* gene was obtained from the genome annotation file of *A. mongholicus*. Its corresponding chromosome physical location was mapped using TBtools software, and the genes were named according to the order of their chromosome locations. The evolution relationships within the species of *A. mongholicus* and with the other five species were analyzed using MCScanX.^24^ The Advanced Circos function of TBtools software was used to visualize *AmYABBY* gene-replication events, and the Dual Systeny Plot function was used to visualize the covariate relationships between *A. mongholicus* and other species.

### Phylogenetic analysis of AmYABBY and AtYABBY proteins

The *A. thaliana* YABBY protein sequences were downloaded from the TAIR database (https://www.arabidopsis.org/). The *O. sativa* and *Glycine max* YABBY protein sequences were downloaded from the Phytozome (https://phytozome-next.jgi.doe.gov/). The full-length protein sequences of AmYABBY and AtYABBY were aligned by multiple sequence comparison using MEGA-Ⅹ11 software,^25^ and the phylogenetic tree was constructed using the neighbor-joining (NJ) method with the bootstrap replicated 1000 times. The AmYABBY proteins were divided into different subfamilies based on the classification of *A. thaliana* YABBY proteins. AmYABBY proteins were categorized into subfamilies based on the *A. thaliana* YABBY protein classification and beautified through the Evolview website (www://evolgenius.info/evolview-v3/).

### AmYABBY gene motif, structure, and cis-element analysis

DNAMAN software was used to analyze the conserved domain of the AmYABBY protein. Motif analysis of *AmYABBY* gene family members was performed using the MEME website (http://meme-suite.org/tools/meme)^26^ with parameters set to the Classic mode, ZOOPS, and number of motifs set to ten. All *AmYABBY* gene upstream 2000 bp sequences were used as the promoter region and submitted to the PlantCRAE website (http://bioinformatics.psb.ugent.be/webtools/plantcare/html/)^27^ to analyze their *cis*-element composition. TBtools software was applied to visualize the phylogeny, motif, conserved structural domains, gene structure, and *cis*-element composition of the *AmYABBY* gene.

### Analysis of RNA-Seq data and real-time quantitative PCR (qRT-PCR) validation

The transcriptome data of roots, stems, and leaves of *A. mongholicus* used in this study were measured by our group. The expression of each *AmYABBY* gene was quantified in terms of reads per kilobase of transcription per million mapped reads (FPKM) values. All FPKM values were processed using log_2_(FPKM +1) log transformation, and heatmaps were generated using Tbtools software.Total RNA was extracted from the roots, stems, and leaves of *A. mongholicus* using the Plant Total RNA Extraction Kit Plant Total RNA Extraction Kit (Tiangen Biochemical Technology Co., Ltd., Beijing, China). Then, the first strand of cDNA was synthesized by reverse transcription using a cDNA First Strand Synthesis Kit (Msunflowers Biotech Co., Ltd., Beijing, China) and then synthesized using a Real-Time Fluorescence PCR Kit (Vazyme Biotech Co. Ltd., Nanjing, China) to perform qRT-PCR experiments. The AriaMx real-time PCR system (Agilent Technologies) was used for qRT-PCR experiments. The *18s* gene was used as the internal reference gene, and three technical replicates were set up for each experiment. The 2^−ΔΔCt^ method^28^ was used to calculate the relative gene expression in each tissue. Primer Premier (version 5.0) software was used to design qRT-PCR specific primers. Then, GraphPad prism (v8.0.2) software was used to plot relative gene expression histograms and analyze them via t-test. The primer sequences used for qRT-PCR can be found in Supplementary Table S1 (Table S1).

## Results

### Identification and physicochemical property analysis of AmYABBY genes

After removing redundant and incomplete sequences with structural domains, seven *AmYABBY* genes were finally identified (Table 1, File S1). In subsequent analyses, we named these genes *AmYABBY1* to *AmYABBY7* based on the gene’s position in the chromosome or genome. Detailed physicochemical property analyses were subsequently performed, which indicated that the amino acid lengths of the AmYABBY proteins ranged from 750 (AmYABBY5) to 495 (AmYABBY1). The predicted molecular weight of AmYABBY proteins ranges from 62,531.52 Da −41810.41 Da, and the isoelectric point is 5.10–5.18. The mean hydrophilicity of all AmYABBY proteins is less than 0, and probably all of them are hydrophilic.

**Table 1.** Physicochemical properties and secondary structure analysis of *AmYABBY* gene family.

Gene ID	Gene name	Number of amino acids	Mw/Da	Mw/kDa	GRAVY	Location	Alpha helix (%)	Beta turn (%)	Extent strand (%)	Random coil (%)
Am01G016200.1	*AmYABBY1*	495	41810.41	5.18	0.893	Nucleus	18.9	0	11.59	69.51
Am01G025050.1	*AmYABBY2*	645	55727.09	5.1	0.961	Nucleus	21.03	0	21.03	68.22
Am01G030550.1	*AmYABBY3*	561	46627.34	5.17	0.783	Nucleus	22.58	0	12.9	64.52
Am01G038910.1	*AmYABBY4*	543	45175.83	5.18	0.856	Nucleus	18.89	0	12.78	68.33
Am04G012270.1	*AmYABBY5*	750	62531.52	5.16	0.743	Nucleus	30.52	0	12.05	57.43
Am04G015730.1	*AmYABBY6*	519	44127.39	5.16	0.967	Nucleus	15.12	0	12.79	72.09
Am05G019820.1	*AmYABBY7*	528	43975.63	5.18	0.882	Nucleus	17.14	0	13.14	69.71

According to the predicted data of secondary structure, the values of α-helices of the proteins encoded by the *YABBY* gene were all greater than 0.1512. Among them, the α-helices of *AmYABBY1*, AmYABBY4, and AmYABBY7 were very close to each other, and the α-helices of AmYABBY2 and AmYABBY3 showed similarity. The results in terms of β-turning angle showed that AmYABBY1 to AmYABBY7 proteins all had a get-turn angle degree of 0. The elongated chains were 0.4735 to 0.6223, of which AmYABBY3 and AmYABBY4, AmYABBY5 and AmYABBY6 had similar elongation lengths, respectively. The Irregular curls were 0.5743 ~ 0.7209, with similar degrees of irregular curls for AmYABBY1, AmYABBY2, AmYABBY4, and AmYABBY7 (Table 1).

The tertiary structures of AmYABBY4, AmYABBY7, amyabby1, and AmYABBY6 proteins were similar, and the tertiary structures of AmYABBY2, AmYABBY3, AmYABBY4, and AmYABBY5 proteins were consistent. The results showed that some gene family members had the same tertiary protein structure (Supplementary Figure S1).

### Chromosomal localization and fragment-duplication events of AmYABBY genes

**Figure 1.**
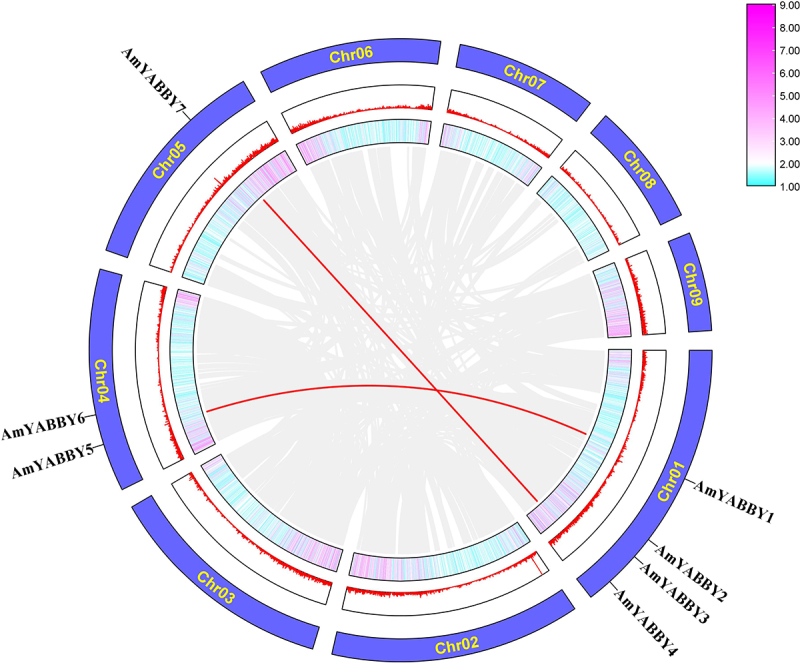
Chromosome distribution and fragment-replication event analysis of the *AmYABBY* gene family. Red-line generations indicate tandem duplication relationships between *AmYABBY* genes. The two outer circles represent gene density information: pink indicates high gene density, while blue indicates low gene density.

The distribution of *AmYABBY* genes on the chromosomes of *A. mongholicus* did not have a clear pattern. Seven *AmYABBY* genes were unevenly distributed on the three chromosomes of *A. mongholicus*. Chromosome one contained the largest number of *AmYABBY* gene family members (*AmYABBY1*, *AmYABBY2*, *AmYABBY3*, and *AmYABBY4*), and chromosome five contained the smallest number of *AmYABBY* genes (Figure 1). To understand the expansion of the *AmYABBY* gene family, an investigation of the gene-duplication event in *A. mongholicus* was carried out. Two pairs of *AmYABBY* genes in the *AmYABBY* gene family were generated by segmental duplication: *AmYABBY1 AmYABBY5* and *AmYABBY4*， *AmYABBY7*.

### *Phylogenetic analysis of the YABBY gene family in* A. mongholicus

**Figure 2.**
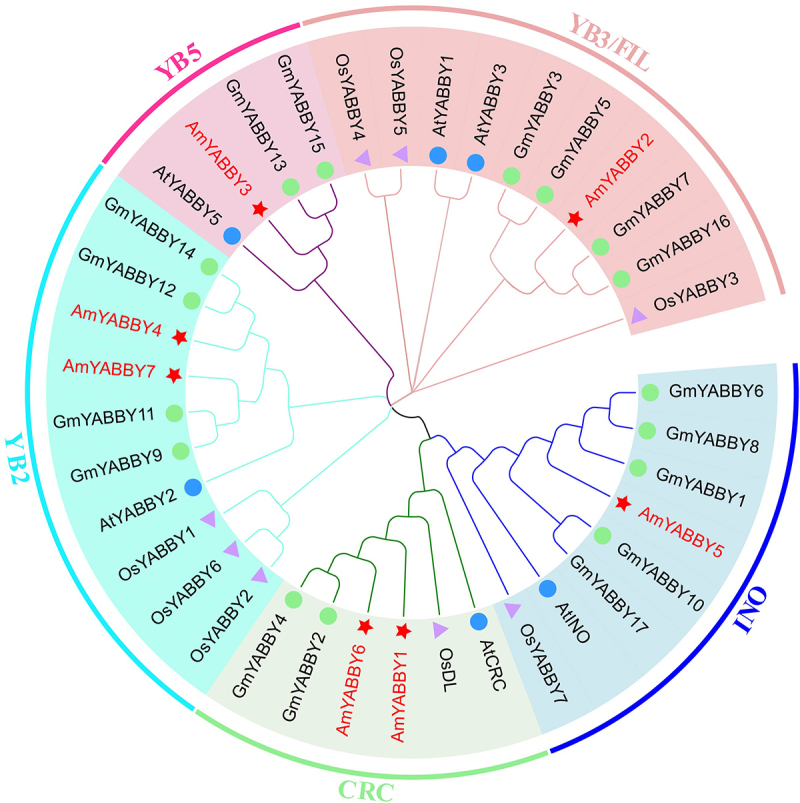
Phylogenetic tree of YABBY proteins in *A. mongholicus* and *A. thaliana*. Red stars represent the *A. mongholicus* YABBY protein, blue circles represent the *A. thaliana* YABBY protein, purple triangles represent rice proteins, and blue-green circles represent soy proteins.

The amino acid sequences of *A. thaliana, O. sativa* and *G. max YABBY* family members were downloaded to explore the evolutionary relationship and classification of the *AmYABBY* gene family. We constructed a neighbor-joining phylogenetic tree using the YABBY protein sequences of *A. mongholicus*, *O. sativa*, *G. max* and *A. thaliana*. Phylogenetic analysis showed that seven AmYABBY proteins were divided into five subfamilies, including CRC containing AmYABBY1 and AmYABBY6, YB5 containing AmYABBY3, INO containing AmYABBY5, YB2 containing AmYABBY4 and YABBY7, and YB3/FIL containing AmYABBY1. The results of phylogenetic tree analysis showed that the AmYABBY Proteins of *A. mongholicus* and *A. thaliana* had high homology in each cluster (Figure 2).

### AmYABBY gene structure and cis-element analysis

**Figure 3.**
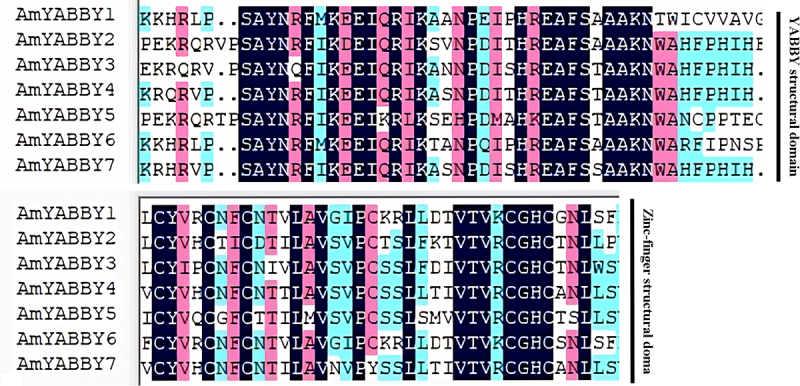
Alignment of conserved sequences of AmYABBY proteins, which are the YABBY conserved domain and zinc finger domain, respectively.

Sequence alignment of *AmYABBY* family members was performed. The alignment results showed that both *AmYABBY1-AmYABBY7* proteins contained a *YABBY* domain and a zinc finger domain (Figure 3). There are conserved amino acids such as cysteine, leucine, proline, and glycine.

**Figure 4.**
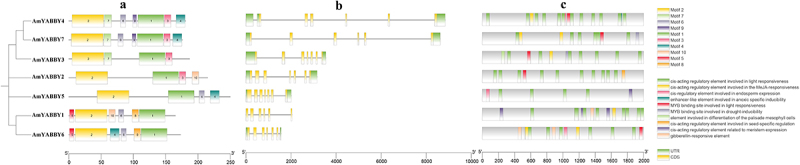
Analysis of gene motif, gene structure, and *cis*-element of *AmYABBY* gene family. (a) AmYABBY protein motif composition; different colors represent different motifs. (b) *AmYABBY* gene structure. CDS: coding sequence, UTR: in the untranslated region, lines denote introns. (c) The *cis*-acting element contained in the *AmYABBY* gene family.

To explore the similarity of AmYABBY protein structures, 10 conserved motifs were identified using the MEME tool. These conserved motifs ranged from 7 to 50 amino acids in length, with at least 4 to 7 conserved motifs distributed among 7 AmYABBY proteins (Figure 4a). All sequences had motif1 (YABBY domain) and motif2 (zinc finger domain) (Supplementary Figure S2), which were further analyzed as the zinc finger structural domain and the YABBY structural domain, respectively. Evolutionarily related members have similar motif composition. For example, all members of the YB2 subfamily have seven identical motifs.

To further understand the evolution of *AmYABBY* genes, we compared the sequences of *AmYABBY* genes in *A. mongholicus* and analyzed their coding regions and introns (Figure 4b). All members contained introns, and the number of introns was distributed in the range of five to six. As expected, the number of introns was relatively conserved among members of the same subfamily, the YB2 subfamily and the CRC subfamily, which both featured five to six introns.

To investigate the potential transcriptional regulatory role of *AmYABBY*, *cis*-elements were extracted from the PlantCARE database in this study using a 2000 bp upstream element of the *AmYABBY* gene as the promoter region. A total of 95 valuable *cis*-elements were identified in the promoter regions of 7 *AmYABBY* genes, and these *cis*-elements can be broadly categorized into 3 groups (Figure 4c). Elements related to plant growth and development included light response, meristematic tissue expression, endosperm expression, seed-specific regulatory response elements, and fenestrated chloroplast differentiation. *Cis*-elements associated with hormones include gibberellin and methyl jasmonate response elements. Those with involvement in the abiotic stress-related response include *cis*-elements that bind sites with MYB genes to participate in drought response. This suggests that *AmMYB* genes may regulate *AmYABBY* genes to form a regulatory network for different biological functions.

### Synteny analysis of AmYABBY genes

**Figure 5.**
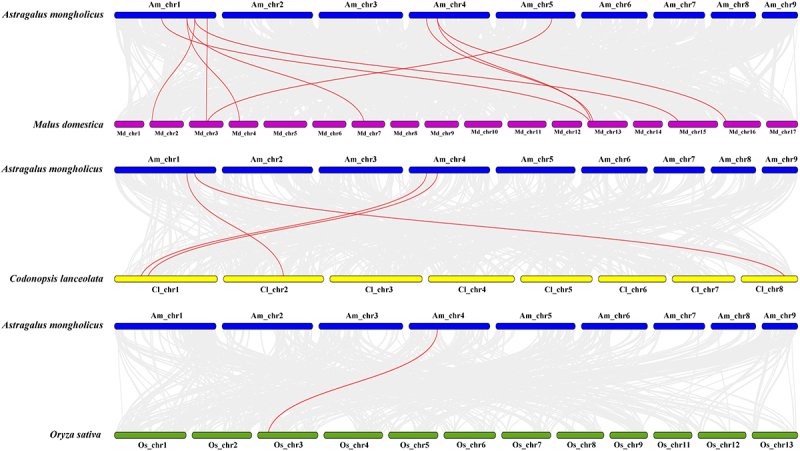
Genomic covariance of *A. mongholicus* with *M. domestica* and *C. lanceolata* in *O. sativa*. The red line indicates the co-linearity relationship of the *YABBY* gene from top to bottom. Blue, purple, yellow, and green colors represent *A. mongholicus*, *M. domestica*, and *C. lanceolata* in *O. sativa*, respectively.

In order to further understand the evolutionary relationship of the *YABBY* gene family among different species, we constructed a covariance between *A. mongholicus* and two dicotyledons (*M. domestica* and *C. lanceolata*) and one monocotyledon (*O.sativa*). The results showed that *AmYABBY* and *MdYABBY* were the most closely co-related with ten gene pairs, *AmYABBY* and *ClYABBY* were the most closely co-related with four pairs, and *AmYABBY* and *OsYABBY* were poorly co-related with only one pair. These results indicate that most of these homologous *YABBY* gene pairs occurred after the differentiation of dicotyledonous and monocotyledonous plants (Figure 5).

### *Expression pattern of AmYABBY genes in different tissues of* A. mongholicus *qRT-PCR verification*

**Figure 6.**
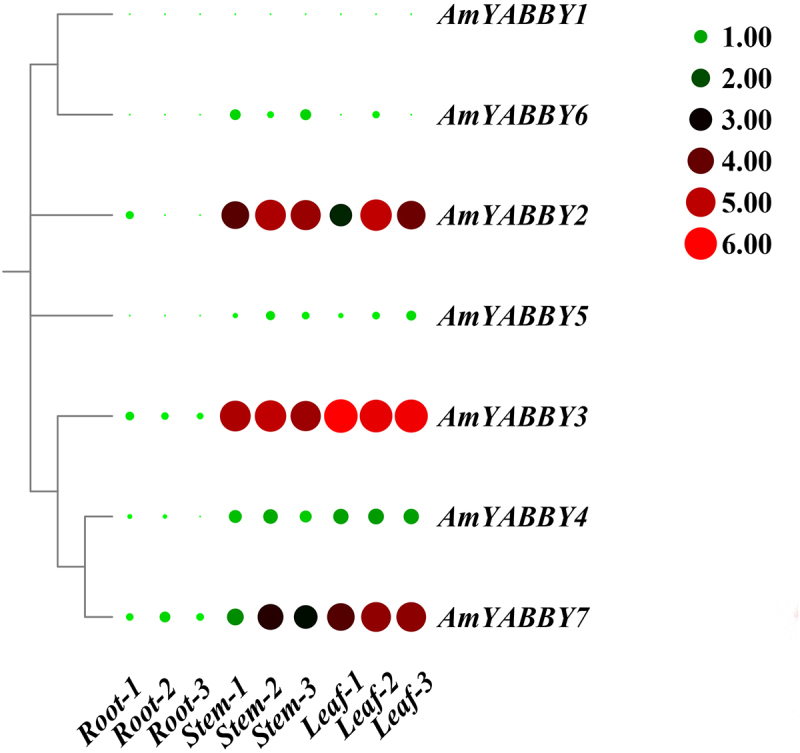
Heat map of *YABBY* gene expression pattern in roots, stems, and leaves of *A. mongholicus*.

In order to investigate the expression pattern of *AmYABBY* genes in different tissues, the gene expression levels of seven *AmYABBY* genes were analyzed based on the stem transcriptome data of leaves and roots of *A. mongholicus*. The results showed that five genes were expressed in stems and leaves (RPKM >0.5), and all the genes were expressed at low levels in roots. Two genes were expressed at very high levels in leaves (*AmYABBY2* and *AmYABBY3*), and four genes were expressed at very high levels in leaves (*AmYABBY1*, *AmYABBY2*, *AmYABBY3*, and *AmYABBY7*). *AmYABBY1*, *AmYABBY3*, and *AmYABBY7* were expressed in all tissue parts, and these genes may be involved in the entire growth and development cycle of *A. mongholicus*. Two genes (*AmYABBY5* and *AmYABBY6*) were not expressed in all tissue sites (RPKM <0.5) and may be pseudogenes or require specific conditions to activate expression (Figure 6, Table S2).

**Figure 7.**
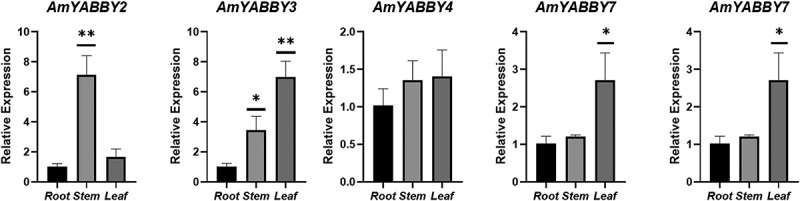
Relative expression levels of 5 *AmYABBY* genes in different tissue sections of *A. mongholicus* detected by qRT-PCR.

Based on the transcriptome data, these five *AmYABBY* genes were selected as candidate genes for qRT-PCR analysis in this study. The results showed that the expression patterns of the five candidate genes were basically consistent with the expression trends obtained from the RNA-seq data. Notably, *AmYABBY1* and *AmYABBY2* had higher expression in stems, and *AmYABBY3* and *AmYABBY7* had higher expression levels in leaves. All of the above indicated that these five *AmYABBY* genes might be closely related to the growth and development of *A. mongholicus* (Figure 7).

## Discussion

*YABBY* genes are a unique gene family in plants, which are more thoroughly studied in the field of plants.^29^ The *YABBY* gene family is not only important for the regulation of plant growth and development but also involved in the regulation of plant secondary metabolism and abiotic stresses^30^; for example, overexpression of *AaYABBY5* in *Artemisia annua* significantly enhanced the content of artemisinin, and *MsYABBY5* expression also affects the synthesis of monoterpenes and other terpene compounds in *Mentha spicata*.^31^ The polar expression of the *YABBY* family is more conserved in dicotyledonous plants, but there is a greater divergence in monocotyledonous plants.^32^ The *YABBY* gene family has been heavily studied in various plants; however, it has not yet been studied in *A. mongholicus*. Therefore, in order to obtain a comprehensive understanding of the basic characteristics of the *A. mongholicus* gene family, we analyzed seven *YABBY* gene family members from the *A. mongholicus* genome. In this study, based on genome-wide data, seven *AmYABBY* genes were identified, and their chromosomal distribution, phylogenetic relationships, motif prediction, gene structure, anterograde element prediction, and expression patterns were comprehensively analyzed. This study provides new insight into further study on the growth and development of the YABBY gene in *A. mongholicus* and its process of molecular mechanism regulation.

In this study, a total of seven *AmYABBY* genes were identified through bioinformatics methods, which is in line with the majority of the previous reports, such as six on *A.thaliana*, six on *Platycodon grandiflorus*, six on *Vitis vinifera*,^33^ and six on *Punica granatum*.^34^ Further analysis revealed that all *AmYABBY* genes contained zinc finger structural domains and *YABBY* structural domains, indicating that the identification results were accurate, reliable, and consistent with those of other plant species.^35^ Physicochemical characterization showed that the average value of hydrophobicity of all *AmYABBY* proteins was greater than 0, suggesting that *AmYABBY* may be hydrophilic proteins which is the same as the identification results of *Nelumbo nucifera*.^36^ Hydrophilic proteins can favor plant resistance to abiotic stresses, which may also be the reason why *YABBY* genes can respond to abiotic stresses. The secondary structures of proteins are mainly composed of four types of spatial structures: the α-helix, β-fold, irregular coil, and extended chain. The secondary structures of proteins of seven genes in the *AmYABBY* gene family are mainly formed by a random coil. The tertiary structures of proteins of *AmYABBY4* and *AmYABBY7* genes are similar.

To investigate the evolutionary relationship of the *AmYABBY* gene family, we constructed a phylogenetic tree using *A. mongholicus* and rice and identified five subfamilies. This is consistent with the results of the developmental tree constructed using *Platycodon grandiflorus*. *YABBY1* and *YABBY6* belong to the CRC subfamily, and various transcription factors in the CRC subfamily can regulate the growth and development process of plants, including the longitudinal and radial growth of the pistil, the influence on the meristem of flowers, the growth of fruit mediastinum, and the formation of carpel wall polarity.^37^ *YABBY3* belongs to the YB5 subfamily, indicating that it can regulate the development of lateral organ polarity, edge formation, leaf maturation, and the stem apical meristem and leaf sequence.^38^ *YABBY1* and *YABBY6* are part of the CRC class, which plays a regulatory role in nectary development in the carpel and core dicots of angiosperms.^39^ *AmYABBY5* is part of INO, which may be involved in the development of the outer integument of ovule*s*.^40^ *AmYABBY4* and *AmYABBY7* are part of YB2, which may be involved in the direction of cell differentiation at the distal end of the outer organ. *AmYABBY2* is part of YB3/FIA, so this gene may has the ability to regulate the formation of plant floral organs and maintain and manage the meristem of floral order. In addition, genes in the same subfamily share the same gene structure and conserved motifs; for example, *AmYABBY4*, *AmYABBY7*, *AmYABBY1*, and *AmYABBY6* are evolutionarily close, indicating that they may have the same function. The consistent number of conserved motifs and gene structures suggests that the two genes may have been generated through gene replication during species evolution.

*Cis*-elements in the promoter region play a key role in regulating gene expression.^41^ For example, 2000 bp upstream of the promoter, several responding elements of *AmYABBY* genes were identified. These can be divided into three categories: light response elements, hormone response elements, and abiotic stress response elements. The *AmYABBY* gene may be closely related to plant hormone signal transduction and plant stress response, especially hormone-induced elements such as gibberellin, abscisic acid, and methyl jasmonate. Expression analysis showed that *AmYABBY3* (YB5 class) genes were highly expressed in leaves, proving that YB5 is crucial for leaf growth and development. The RT-PCR results indicate that *AmYABBY* is highly expressed in stems and leaves. Generally speaking, homologous genes have similar expression patterns, but the expression patterns of *AmYABBY1* and *AmYABBY6* are completely different. It is speculated that the two have undergone functional differentiation due to different expression patterns during evolution, resulting in dissimilar functions produced by homologous genes. Genes with high expression and specific expression may play a role in corresponding tissue parts, and some genes may also undergo functional redundancy.

## Conclusions

Seven *AmYABBY* genes were successfully identified in this study, which were divided into five subfamilies with six or seven introns. In the upstream 2000 bp position of the promoter, we found multiple light-responsive and hormone-responsive elements, suggesting that the *YABBY* gene is useful for expression in various plants. The high expression of *AmYABBY* genes in leaves and stems proves that they play an integral role in the growth and development of *A. mongholicus*. Therefore, the results of this study help to further understand the role of *AmYABBY* genes, lay a solid foundation for the taxonomic and functional studies of *YABBY* in other plants, and provide an important data basis for future studies on the growth, developmental regulation, and quality improvement of *A. mongholicus*.

## Supplementary Material

Supplementary File S1.doc

Supplementary Table S1.doc

Supplementary Figure.doc

Supplementary Table S2.doc

## Data Availability

The transcriptome data were deposited at the NCBI database under accession number PRJNA1064679.
