# Supplementary material for: Genome-wide identification of YABBY gene family and its expression pattern analysis in Astragalus mongholicus
Source: Plant Signal Behav. 2024 May 22;19(1):2355740. doi: 10.1080/15592324.2024.2355740 (PMC11123558; doi:10.1080/15592324.2024.2355740)
Supplement: Supplementary File S1.doc [file KPSB_A_2355740_SM2844.doc]

>Am01G038910.1

MSMDMNMMANERVCYVHCNFCNTTLAVSVPCSSLLTIVTVRCGHCANLLSVNMGASFQTFPPQDPQLLKQHLTSTQEPSCKELGSSSSKCKAFEPEQHRIPPIRPPEKRQRVPSAYNRFIKEEIQRIKASNPDITHREAFSTAAKNWAHFPHIHFGLKLDGSKQAKLDQGDATQKANGFY*

>Am01G030550.1

MSSFSIDVAPEQLCYIPCNFCNIVLAVSVPCSSLFDIVTVRCGHCTNLWSVNMAAAFQSLSWQDVQGPGHCNPEYRIHTGSTSKCNDRVPVRAPTTHVTQERVVNRPPEKRQRVPSAYNQFIKEEIQRIKANNPDISHREAFSTAAKNWAHFPHIHFGLMLETNNQAKMENVSEKRLMSRAALLNK*

>Am01G025050.1

MSSSSTTLSLDHLPPSEQLCYVHCTICDTILAVSVPCTSLFKTVTVRCGHCTNLLPVNMRGLLLPSSPNQFHLGHSFFSPSHNLLQEEMPNPTPNFLMNHTNITPNDFSMPARTVTDELPRPPIINRPPEKRQRVPSAYNRFIKDEIQRIKSVNPDITHREAFSAAAKNWAHFPHIHFGLMPDQTMKKTNLCQQEGDEVLMNYASAANVGVSPY*

>Am01G016200.1

MNHEEKVTMDLVPPSDHLCYVRCNFCNTVLAVGIPCKRLLDTVTVKCGHCGNLSFLSTRPPLNQNHGVEVDHSLNFKKGQASSSSSSSSATSGEPMSPKAAPFVVKPPEKKHRLPSAYNRFMKEEIQRIKAANPEIPHREAFSAAAKNTWICVVAVGKTTYKIK*

>Am04G012270.1

MMIDYECSTSTINKCFTVSKQLSLFFLFLSQTHVLNTLMSTLNHLFDLPEQICYVQCGFCTTILMVSVPCSSLSMVVTVRCGHCTSLLSVNMMKASFVPFHLLASLTNLEPKESSPDEDAIKALNCNSASMMTYSDCEDDDVIPISNVVNKPPEKRQRTPSAYNRFIKEEIKRLKSEHPDMAHKEAFSTAAKNWANCPPTECKGDEESCSQIDQLVDLDFNDVEVNEEGQGFRGRKVARNSILERTPFE*

>Am04G015730.1

MNHEEKVTMDFVPPSDHFCYVRCNFCNTVLAVGIPCKRLLDTVTVKCGHCSNLSFLTTRPPTSQNQTVDHTLSLQGFYGVKKGQGPSSSSSPTRSSESVSPKAAPFVVKPPEKKHRLPSAYNRFMKEEIQRIKTANPQIPHREAFSAAAKNWARFIPNSPTNSIASSKTNIE*

>Am05G019820.1

MDMNHMMATERVCYVHCNFCNTILAVNVPYSSLLTIVTVRCGHCANLLSVNMAASLQPFPPQLPQKQQLILEEPSMKELGSSSKIAAFEAVEQHEPPRIPPIRPTEKRHRVPSAYNRFIKEEIQRIKASNPDISHREAFSSAAKNWAHFPHIHFGKQSKLDLDHGEETEKSHGFY*
