# Supplementary material for: Genome-wide identification of YABBY gene family and its expression pattern analysis in Astragalus mongholicus
Source: Plant Signal Behav. 2024 May 22;19(1):2355740. doi: 10.1080/15592324.2024.2355740 (PMC11123558; doi:10.1080/15592324.2024.2355740)
Supplement: Supplementary Table S1.doc [file KPSB_A_2355740_SM2843.doc]

| Gene Name | Primer |
| --- | --- |
| qAmYABBY1-F | TCACCTCTGCTACGTTCGTT |
| qAmYABBY1-R | AGGGAGGTCTAGTGCTGAGA |
| qAmYABBY2-F | CACTAACCTCCTGCCAGTCA |
| qAmYABBY2-R | GAGTTGGGTTTGGCATCTCC |
| qAmYABBY3-F | GGGCACTGCACCAATCTATG |
| qAmYABBY3-R | GGGCACTGCACCAATCTATG |
| qAmYABBY4-F | TGATGGCAAACGAACGTGTT |
| qAmYABBY4-R | ATGTCCGCATCTAACCGTCA |
| qAmYABBY7-F | TCCTCCTCAACTCCCTCAGA |
| qAmYABBY7-R | AGGACGAATGGGAGGAATCC |
