# Supplementary material for: Genome-wide identification of YABBY gene family and its expression pattern analysis in Astragalus mongholicus
Source: Plant Signal Behav. 2024 May 22;19(1):2355740. doi: 10.1080/15592324.2024.2355740 (PMC11123558; doi:10.1080/15592324.2024.2355740)
Supplement: Supplementary Figure.doc [file KPSB_A_2355740_SM2842.doc]

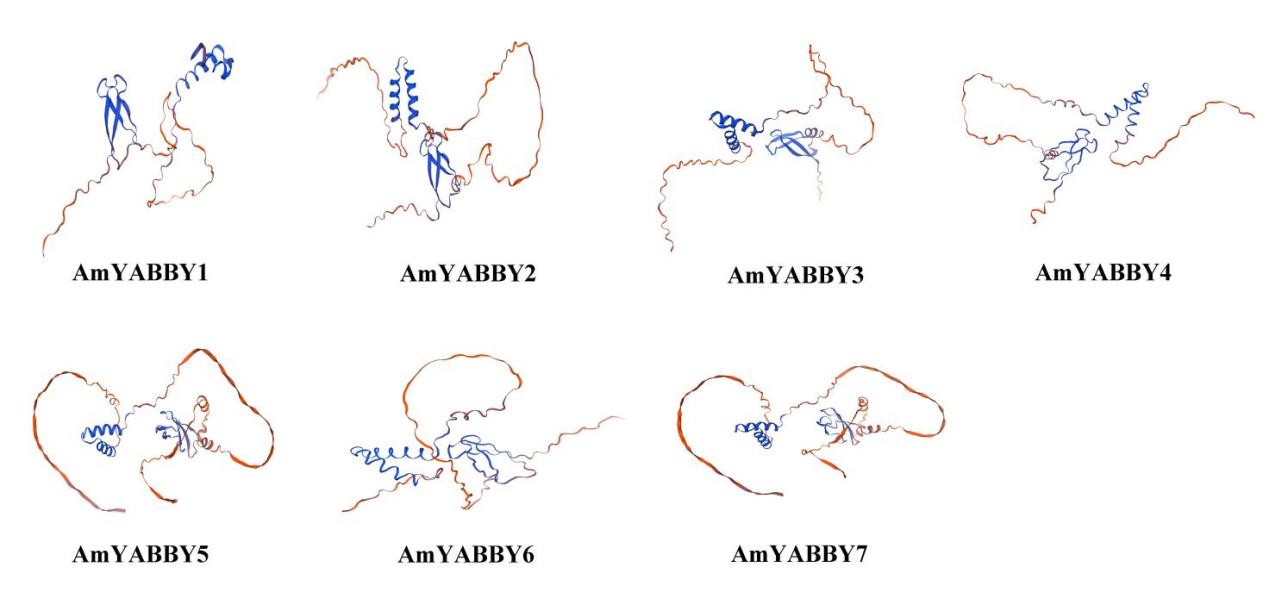


**Supplementary Figure 1. Tertiary structure of AmYABY protein.**

**
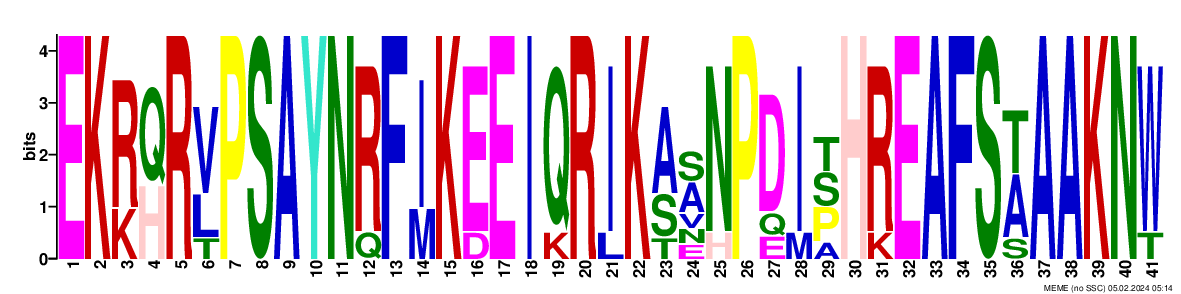

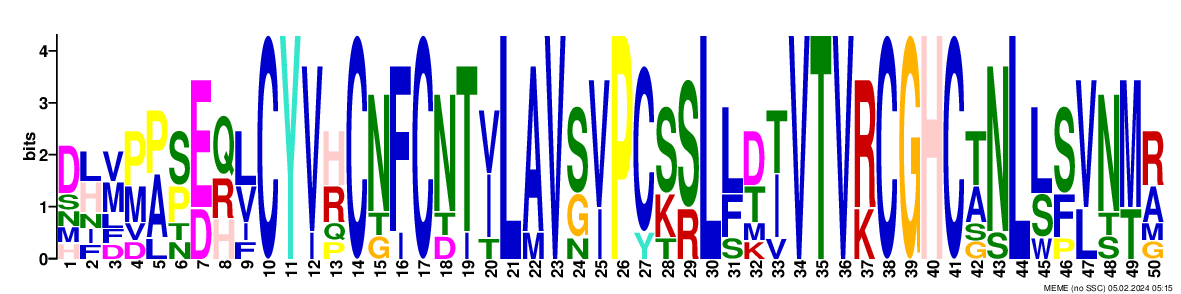
**

**Supplementary Figure 2. Motif1 and Motif2**
