# Supplementary material for: Genome-wide identification of YABBY gene family and its expression pattern analysis in Astragalus mongholicus
Source: Plant Signal Behav. 2024 May 22;19(1):2355740. doi: 10.1080/15592324.2024.2355740 (PMC11123558; doi:10.1080/15592324.2024.2355740)
Supplement: Supplementary Table S2.doc [file KPSB_A_2355740_SM2841.doc]

|  | Root-1 | Root-2 | Root-3 | Stem-1 | Stem-2 | Stem-3 | leaf-1 | leaf-2 | leaf-3 |
| --- | --- | --- | --- | --- | --- | --- | --- | --- | --- |
| AmYABBY1 | 0.09105625 | 0.027350857 | 0.028815068 | 9.06564503 | 13.16871928 | 10.41482623 | 4.986993802 | 16.89658802 | 11.13517464 |
| AmYABBY2 | 0.138362817 | 0 | 0 | 16.96753393 | 26.15204971 | 28.85994401 | 4.677711682 | 42.31879251 | 19.02866021 |
| AmYABBY3 | 0.189822515 | 0.136842232 | 0.108125995 | 37.92347023 | 34.34549224 | 33.63196104 | 63.09626411 | 63.94905887 | 61.85506648 |
| AmYABBY4 | 0.025318714 | 0 | 0 | 0.593315949 | 0.739578402 | 0.50121387 | 0.941560836 | 1.429489115 | 1.007314085 |
| AmYABBY5 | 0 | 0 | 0 | 0.028421469 | 0.061477729 | 0.088535127 | 0 | 0.102932781 | 0.0821231 |
| AmYABBY6 | 0 | 0 | 0 | 0.270225735 | 0.073064768 | 0.315665458 | 0 | 0.152916347 | 0 |
| AmYABBY7 | 0.103772113 | 0.280533258 | 0.131356189 | 1.662406159 | 7.771319318 | 6.505256781 | 13.08589843 | 26.38393854 | 22.89261625 |
